# Royal College of Psychiatrists - UK Workforce Planning Project

**DOI:** 10.1192/j.eurpsy.2026.10234

**Published:** 2026-07-31

**Authors:** D. S. Mullin, J. Parker, J. Anslow, B. R. Underwood, M. Bhat

**Affiliations:** 1 Division of Psychiatry, University of Edinburgh, Edinburgh; 2 Avon and Wiltshire Mental Health Partnership NHS Trust, Bristol; 3 South London and Maudsley NHS Foundation Trust, London; 4 Cambridgeshire and Peterborough NHS Foundation Trust; 5 Department of Psychiatry, University of Cambridge, Cambridge; 6 Psychiatry, Kent and Medway Mental Health NHS Trust, Kent, United Kingdom

## Abstract

**Introduction:**

The UK population is ageing rapidly, with a large cohort currently aged 55–65 soon to enter the 65+ age band, increasing demand for old age psychiatry services. Older adults have elevated rates of psychiatric comorbidity, particularly in the context of frailty and dementia (Age UK, 2023). Recent national surveys also indicate rising prevalence of common mental health conditions in younger and middle-aged adults (NHS Digital, 2023), suggesting that future cohorts entering older age may have higher psychiatric morbidity.

**Objectives:**

This project, commissioned by the Royal College of Psychiatrists’ Faculty of Old Age, aims to guide psychiatrist workforce planning by assessing current service capacity and forecasting future needs, thereby informing College and national policy.

**Methods:**

The work comprises three strands: (1) mapping the distribution of the old age psychiatry workforce across the UK, including consultant full-time equivalents (FTEs), regional variation, and training numbers; (2) correlating workforce supply with demographic and epidemiological trends to identify misalignment between need and provision; and (3) modelling future workforce supply and demand, taking into account training patterns, projected retirements, and service use models. Regular supervision is provided by the Faculty Chair and Vice Chair, with stakeholder engagement ensuring applicability to service planning.

**Results:**

Preliminary analyses suggest that demand for old age psychiatry will outpace consultant growth, exacerbating existing regional disparities. Consultant provision in old age psychiatry is insufficient: current estimates indicate approximately one consultant per 23,000 adults aged 65+, projected to deteriorate markedly over the next 10 years without corrective action. This work commenced in August 2025 and is ongoing; full analyses across the three workstreams will be completed by March 2026, with comprehensive results available for presentation at the EPA Congress.

**Conclusions:**

Urgent workforce planning is required to expand training capacity, enhance retention, and align resources with projected demographic and morbidity trends. The findings will be disseminated through publication and national conference presentation, contributing to evidence-based workforce policy for the specialty.

**Disclosure of Interest:**

None Declared

